# Functions and Regulation of Endogenous Retrovirus Elements during Zygotic Genome Activation: Implications for Improving Somatic Cell Nuclear Transfer Efficiency

**DOI:** 10.3390/biom11060829

**Published:** 2021-06-02

**Authors:** Bo Fu, Hong Ma, Di Liu

**Affiliations:** 1 Institute of Animal Husbandry, HeiLongJiang Academy of Agricultural Sciences, Harbin 150086, China; fubohao810@163.com (B.F.); mahong197400@163.com (H.M.); 2 Key Laboratory of Combining Farming and Animal Husbandry, Ministry of Agriculture and Rural Affairs, Harbin 150086, China

**Keywords:** preimplantation embryo, endogenous retroviruses, zygotic genome activation, double homeobox, somatic cell nuclear transfer

## Abstract

Endogenous retroviruses (ERVs), previously viewed as deleterious relics of ancestral retrovirus infections, are silenced in the vast majority of cells to minimize the risk of retrotransposition. Counterintuitively, bursts of ERV transcription usually occur during maternal-to-zygotic transition (MZT) in preimplantation embryos; this is regarded as a major landmark event in the zygotic genome activation (ZGA) process, indicating that ERVs play an active part in ZGA. Evolutionarily, the interaction between ERVs and hosts is mutually beneficial. The endogenization of retrovirus sequences rewires the gene regulatory network during ZGA, and ERV repression may lower germline fitness. Unfortunately, owing to various limitations of somatic cell nuclear transfer (SCNT) technology, both developmental arrest and ZGA abnormalities occur in a high percentage of cloned embryos, accompanied by ERV silencing, which may be caused by the activation failure of upstream ERV inducers. In this review, we discuss the functions and regulation of ERVs during the ZGA process and the feasibility of temporal control over ERVs in cloned embryos via exogenous double homeobox (DUX). We hypothesize that further accurate characterization of the ERV-rewired gene regulatory network during ZGA may provide a novel perspective on the development of preimplantation embryos.

## 1. Introduction

Following fertilization and activation, the maternal-to-zygotic transition (MZT), which includes global transcriptional and epigenetic changes in the preimplantation embryo, is initiated. During this transition, zygotic genome activation (ZGA) occurs, when the zygote has divided into a two-cell embryo or four-cell embryo (depending on the species). The ZGA proceeds through two main phases, namely minor ZGA and major ZGA. In mice, the minor ZGA occurs between S phase of the one-cell embryo and G1 of the two-cell embryo, while the major ZGA occurs between mid-two-cell stage and late-two-cell stage. Specifically, loosened chromatin structure and promiscuous transcription usually emerge during minor ZGA, whereas the burst of transcription presents during major ZGA [1,2]. The occurrence of developmental arrest depends on the state of ZGA. The ZGA process destroys the maternally inherited transcripts and selectively activates zygotic genes as well as a great number of retrotransposons, especially endogenous retroviruses (ERVs).

Retrotransposons are divided into long terminal repeat (LTR) retrotransposons and non-LTR retrotransposons; ERVs are classified as LTR retrotransposons. ERVs account for approximately 5–15% of genomic content [3,4,5]. The reverse transcription process has been activated for at least 450 million years [6]. The Envelope (*Env*) gene contained in exogenous retroviruses encodes a transmembrane domain that enables these retroviruses to undergo an extracellular infectious phase [3]. Occasionally, numerous exogenous retroviruses infect the germline and integrate into the host germline genome, after which they are vertically inherited in the host population. This process causes the accumulation of ERVs of diverse origins in infected species, ultimately establishing novel ERV families [3,6,7,8]. In theory, ERVs contain coding sequences, such as the genes *Env*, *Gag*, and *Pol*, that are flanked by LTRs, and therefore have the ability to replicate and mobilize, but in reality, most ERVs either lack viral ENV proteins or have truncated or mutated open reading frames (ORFs), which result in non-infective ERVs and non-horizontal transfer between individuals. ERV activation is traditionally viewed as a threat to genomic stability. Once transposable ERVs are constitutively active, the exponential amplification of an ERV-derived element will result in random genome instability in situ and mutations, which may change gene structure and expression. Retrotransposition, which is a hallmark event in advanced cancer stages, often occurs in various pathological conditions [9,10,11,12].

Counterintuitively, a large number of ERVs, previously viewed as parasitic junk sequences [13,14], are preferentially activated during ZGA in preimplantation embryos as well as in a subpopulation of mouse embryonic stem cells. For example, MuERV-L elements, which are ERV-derived elements in mice, are transcribed during ZGA when paternal chromosomes rapidly lose protamine and active demethylation of the male pronucleus occurs [15]. Chimeric transcripts containing elements that originated from the LTR family of ERVs have also been identified in early mouse cleaved embryos [16]. Additionally, Macfarlan et al. identified a subpopulation of embryonic stem cells, known as two-cell-like cells (2CLC), in which murine endogenous retrovirus (MERVL) was transcribed robustly [17].

Owing to the different histories of host exposure to exogenous retroviruses, the genomic content of ERVs varies significantly between species [18]. It remains an open question how such evolutionarily divergent ERV elements can mediate the conserved ZGA process. It is now known that an extensive dynamic change in gene expression patterns is a hallmark of ZGA during epigenomic reprogramming and that ERVs usually take advantage of this developmental epigenomic reprogramming window to evade epigenetic silencing; thus, the failure of transient ERV activation is tightly linked to ZGA defects [19]. Increasing evidence suggests that robust ERV expression may have multiple biological roles in early embryos, such as initiating the synchronous, transient expression of multiple conventional genes, facilitating high-order chromatin assembly, and defending against exogenous viral infection [16,20,21]. ERV activation can even be regarded as a hallmark of totipotency [22]. By activating MERVL, 2CLCs acquire the unique molecular and developmental features of totipotent two-cell-stage embryos [23,24,25]. An ERV loss of function assay applying RNA knockdown also revealed that ERV expression is essential for preimplantation embryo development [26]. What is the driving force that triggers these bursts of species-specific ERV expression, then dominates the conserved ZGA process during preimplantation development? Wide-spread derepression of double homeobox (*Dux*)-containing repeats occurs immediately after fertilization. It is intronless *Dux* family member genes that activate the expression of ERVs and ZGA-related genes and then play an integral role in the ZGA process [27,28,29].

Somatic cells can be reprogrammed to totipotency and then form live, cloned offspring through somatic cell nuclear transfer (SCNT) technology [30]. This technology has great potential for use in animal husbandry and endangered species conservation. Although more than 20 mammalian species have been successfully cloned [31,32], cloning efficiency is still low [33]. Notably, both the transcriptional deregulation of ERVs and severe ZGA defects were observed in the vast majority of cloned embryos [34,35,36,37]. Correspondingly, the cloned embryos also exhibited abnormal DUX transcription [38,39]. Owing to the versatility of ERVs during the ZGA process, transient DUX expression during minor ZGA may provide a novel approach for alleviating ZGA defects in cloned embryos. Currently, research devoted to improving SCNT efficiency through introducing exogenous DUX into cloned embryos, which we discuss further below, is ongoing.

It has been proposed that, during the epigenetic reprogramming window when ERVs evade silencing by their host, the host regulatory pathways are hijacked by ERVs to favor their own transcription and propagation. In practical terms, however, a subset of ERV elements have been co-opted as regulatory elements to rewire the gene expression network through cis- and trans-regulatory mechanisms, thus providing the plasticity of gene expression observed during ZGA. This review aims to outline the multiple roles of ERVs during the ZGA process and the feasibility of regulating ERV activation in a spatiotemporal-specific manner by introducing exogenous DUX into cloned embryos. Precise regulation of ERVs, through their upstream inducers, may allow us to bypass the innate chromatin remodeling abnormalities and ZGA defects of cloned embryos, ultimately facilitating the emergence of new approaches for improving SCNT efficiency.

## 2. ERV Functions during ZGA

Totipotent two-cell mouse embryos, which undergo the normal ZGA process, are characterized by massive transcriptional activation of ERVs [17,23]. The prefabricated coding and regulatory sequences, originally used for viral infection and replication by ERVs, have been occasionally repurposed for cellular function in host. The biological functions played by the activated ERVs at this specific developmental time points are fascinating.

Natural selection provided the selective pressure on ERV LTR elements that initially controlled the proviral genes. Over the course of evolution, recombination between LTRs resulted in the loss of internal ERV genes, which has eliminated the potential fitness cost of expressing ERV genes while keeping the beneficial regulatory effects of LTRs on the host genes. ERVs can be eliminated through non-allelic homologous recombination between their two adjacent LTRs, thus cutting the intervening sequence and retaining a single “solo” LTR. This process has led to a unique landscape of LTRs in the genome where the number of solo LTRs exceeds the number of full-length ERVs [40,41]. LTRs contain cis-regulatory sequences and RNA polymerase II promoters that control transcription initiation [42]. Notably, LTRs perform a variety of functions, such as activating transcription, altering protein-coding sequences, and producing non-coding RNAs during ZGA [43]. A subset of LTRs function as alternative promoters and provide first exons for some conventional host genes or pseudogenes. For example, a massive upregulation of ERV expression, such as that of MuERV-L LTRs, occurs during ZGA, accompanied by an increase in the expression of ERV-adjacent conventional genes. MERVL-LTR elements (e.g., MT2) act as cell-specific robust alternative promoters for promoting nearby two-cell-specific gene expression, with MEVRL and nearby two-cell-specific genes generating chimeric transcripts [16]. A global expression correlation analysis between transposable elements and their nearest genes has shown that expression levels of ERVs, such as ERVK, ERVL-MaLR, and ERVL, are positively correlated with those of their nearest gene, which include *Chka* and *Zfp54*; these genes have been implicated in the regulation of early embryonic development [26]. Transcripts derived from LTR elements have been found in numerous mammalian preimplantation embryos [44,45,46], indicating that this neofunctionalization of ERVs as alterative promoters is driving the exaptation of new promoters for the needs of early development.

Hosts also use ERVs to protect themselves from exogenous retroviral infection. Co-opted ERV-derived proteins are used extensively for viral defense. It has been theorized that ERV-derived products can interfere with any step of viral infection, subsequently restricting virus replication. It is now clear that some interactions between ERV-derived peptides and viral or cellular proteins can control virus replication. For example, ERV-encoded ENV proteins protect host cells from cognate retrovirus invasion by binding cell surface receptors that could otherwise be bound by the ENV proteins of exogenous retroviruses. This competition with exogenous ENV is a phenomenon analogous to super-infection resistance [21]. Additionally, human *Hervk* encodes REC protein, and the ectopic overexpression of REC leads to the upregulation of IFITM1, an interferon-induced viral restriction factor that increases innate antiviral responses; thus, an interferon-mediated innate antiviral pathway is activated through ERV activation in early embryos [47]. Moreover, interactions between ERVs and viral-encoded proteins may drive the rapid adaptive evolution of both viral genes and ERV-derived genes, i.e., allelic diversification of ERV-derived genes may lead to the selection of alleles that expand the range of viruses restricted by these interactions. For example, *Fv1*, identified as a co-opted ERV sequence that is associated with the *Gag* gene in MuERV-L, restricts murine leukemia virus (MLV), exhibiting a strong signature of diversifying selection in mouse populations [48,49].

LincGET, a nuclear long intergenic noncoding RNA (lincRNA), is specifically expressed in two- to four-cell embryos. LincGET is derived from a MERVL locus and functions as a scaffold for the recruitment of transcription factor and splicing factors, which indicated the important for embryonic development [50,51].

ERVs are not just transcribed in early embryos but also have the capacity to be translated and assembled into virus-like particles, which may have a functional role during preimplantation embryonic development. For example, MuERV-L, which encodes canonical retroviral *Gag* and *Pol*, is flanked by 5′ and 3′ LTR (known as MT2_Mm). In general, the level of MuERV-L-Int and MT2_Mm transcripts is higher at the two-cell stage in mouse embryos. Knockdown of MuERV-L by siRNA not only decreases the expression of MuERV-L transcripts but also leads to a significant reduction in MuERV-L Gag protein staining in two-cell embryos, resulting in a mild developmental delay at four to eight-cell stage progression on day 2 and fewer embryos reaching the blastocyst stage on day 4. This phenomenon indicated that MuERV-L Gag protein has a functional role at the two-cell stage development, similar to the role of endogenous LTR viral-like particles in human blastocysts [26,47].

Higher-order chromatin structure plays a key role during regulation of gene expression. Immediately after fertilization, chromatin structure makes a transition from a condensed state to a loosened state. Along with providing chromatin organizer binding sites, ERVs is also involved in the formation of a higher-order chromatin structure. For example, ERVs are enriched in chromatin organizer-binding sites such as CCCTC-binding factor (CTCF) [20]. Given the fact that CTCF play key roles in the establishment of higher-order chromatin structure (including chromatin loop, long-range enhancer-promoter interaction, chromatin insulators, and topological domain border), the chromatin organization function of ERVs could be achieved by binding CTCF [52,53,54,55].

## 3. ERV Activation during ZGA

To broadly co-opt ERVs elements into a regulatory network and minimize the risk of retrotransposition, the expression of ERVs must be regulated precisely during preimplantation development. ERVs are usually transcriptionally silenced in most somatic cells, which can be achieved through DNA methylation and constitutive heterochromatin formation with heterochromatin protein 1 (HP1) as hallmark. Conversely, immediately after fertilization, high level DNA methylation and constitutive heterochromatic structure were not observed at the earliest developmental stages [56], with a great portion of ERVs transcribed in zygote and two-cell stage mouse embryos [16]. Intriguingly, totipotency is restricted to the zygote and each of the blastomeres of the two-cell stage mouse embryo [57,58]. We then wonder what mechanism drove the bursts of ERVs transcription then shaped totipotency of early embryos.

Currently, DUX in mice or DUX4 in humans, a pioneer transcription factor, has been identified as a key regulator of ERVs. After fertilization, *Dux* family genes are transcribed during minor ZGA, then activate the transcription of downstream genes during major ZGA [27,28,29]. Abe et al. also argued that minor ZGA is a prerequisite for major ZGA [2]. DUX in mice (homologous to DUX4 in humans) is a class of double homeodomain transcription factors that is conserved in mammals. The DUX-encoding gene *Dux* is located within tandem repeats consisting of *Dux* paralogs that differ from each other by only a few bases. A DNA-FISH experiment that used *Dux* as a probe revealed that all the *Dux* family genes are present in *Dux* tandem repeats [59,60]. Although oocytes do not express *Dux* family genes, high levels of DUX expression were detected after fertilization in one-cell-stage mouse embryos when minor ZGA occurred, and the DUX expression faded gradually until the late two-cell stage. In addition to Dux, more than a dozen Dux paralogs are also expressed during minor ZGA, which may ensure a sufficient amount of functional DUX [61]. In humans, *Dux4* tandem repeats, located within pericentromeric regions, generally carry characteristic heterochromatin, which prevents their expression in most types of somatic cells. Aberrant DUX expression from these tandem repeats usually causes facioscapulohumeral muscular dystrophy (FSHD) [62,63].

The mechanism by which *Dux* family genes are expressed during minor ZGA is associated with the unique chromatin state of embryos at this stage. Generally, in somatic cells, heterochromatin is formed to repress transcription, then tandem repeats located within macrosatellite repeat regions, is silenced, indicating that the *Dux* family genes located within these tandem repeats are also silenced. In contrast, one-cell-stage mouse embryos have extremely high histone mobility, as demonstrated in fluorescence recovery after photobleaching (FRAP) experiments; furthermore, constitutive heterochromatin, which is normally observed in pericentromeric regions, is still not formed at this stage [64]. Together, these findings suggest that the chromatin structure is loosened dramatically throughout the genome in one-cell-stage mouse embryos, such that one-cell embryos are transcriptionally permissive. Accordingly, this unique loosened chromatin structure results in widespread transcription throughout the whole genome, including intergenic regions such as pericentromeric regions and *Dux* family genes [1,65,66]. Ooga et al. proposed that extremely loosened chromatin may be involved in totipotency and that the loss of this unique chromatin state is associated with differentiation during preimplantation development [64]. Based on a kernel density estimation, over 90% of genes, including retrotransposons, are expressed in embryos at the one-cell stage [67]. This gene expression pattern is not associated with proximal regulated elements but rather with the CpG content around the transcription start site (TSS) [68]. During minor ZGA, the widespread transcription is independent of enhancers, with low transcriptional activity and a short time window for transcription [69], so the expression level of each individual gene is very low [1,67]. Even housekeeping genes are not highly expressed at this stage; there is much lower variation in the gene expression level during the one-cell stage than there is during other stages [67,68]. Fortunately, to produce a sufficient amount of functional DUX, multiple *Dux* paralogs with functions similar to that of DUX are expressed, which consequently increases the expression of DUX target genes such as *Zfp352* and *Zscan4d* [61].

The intronless *Dux* arose via the retrotransposition of an ancestral intron-containing *DUXC*. Owing to the inefficient splicing in one-cell embryos, intronless *Dux* would have had a higher chance to produce functional transcript during minor ZGA compared with genes containing introns. Because DUX-binding sites are located within ERV elements, the targets regulated by DUX should include ERV elements [27,28,29]. For example, DUX initiates MERVL transcription via binding directly to the DUX-recognition motif within the LTR [27,29]. Additionally, DUX4, the human orthologue of murine DUX, can recruit the EP300–CBP complex to local chromatin via its C-terminal domain and then open chromatin around the TSSs of ZGA-related genes (including ERVs), ultimately facilitating access by other transcription factors [70]. Therefore, bursts of ERV expression are always consistent with the highly accessible state of chromatin in mouse embryos during the two-cell stage [71]. Resent research has also demonstrated that the ectopic expression of DUX alone in mouse embryonic stem cells can activate MERVL, accompanied by a “two-cell-like” transcriptional program, which often corresponds with ZGA [27,29]. Indeed, transient overexpression of DUX can significantly increase the expression of MERVL, indicating that DUX acts as a core factor for ZGA by enhancing the expression of ZGA-specific genes such as *Zscan4*, *Zfp352*, and MERVL [72,73]. Given the versatility of ERVs, it seems likely that ancestral DUX proteins strengthened their control over the ZGA process by regulating ERVs [28]. These findings also suggest that ERVs have been co-opted to regulate key developmental processes during ZGA.

Although DUX and DUX4 both activate orthologous ZGA-related genes, such as *Zscan4*, within their respective species, there is only modest sequence conservation between them. Furthermore, when mouse DUX was overexpressed in mouse cells, it activated ZGA-related ERVs, such as MERVL, whereas the overexpression of human DUX4 failed to activate murine MERVL. ChIP–seq data also demonstrated that MERVL elements were targets of DUX but not of DUX4, with a DUX-binding site located within the MERVL elements [28]. Once the more divergent first homeodomain in DUX family proteins binds to the species-specific transcription factor-binding sites located within LTR elements, species-specific ERVs are activated; thus, the divergence of the DUX4 and DUX homeodomains reflects ERV specificity. Meanwhile, the more conserved second homeodomain binds to orthologous ZGA-related genes, such as *Zscan4*, thus activating these genes. The orthologous ZGA-related genes, regulated by DUX family proteins, represent the core ancestral ZGA network, whereas ERV-derived genes reflect species-specificity during ZGA. The above phenomenon indicates that ancestral DUX family proteins, although functionally conserved, have adapted to species-specific activation of ERVs during evolution owing to the divergence of the DNA-binding motif of DUX, ultimately providing a delicate balance between conservation of the ZGA transcriptional program and innovation of ERV-driven transcription.

## 4. Multi-Tiered Regulation of ERVs in Early Embryos

The transient bursts of ERV expression during ZGA do not mean that early embryos have abandoned their control over ERVs. The reactivation of ERVs always poses an inherent risk of potential retrotransposition, especially in early embryos that lose epigenetic silencing during their developmental reprogramming. Along with the activation of ERVs, the suppression of ERVs is also essential for normal embryonic development. Although ERV elements are typically repressed in somatic cells via DNA methylation, preimplantation embryos appear to use alternative mechanisms for minimizing the risk of retrotransposition. ERV expression is under surveillance by multilayered systems that ensure stage-specific ERV expression during preimplantation embryo development.

The inefficient splicing and 3′-processing of nascent transcripts in one-cell embryos may restrict ERV activation. Although the extremely relaxed chromatin structure in one-cell embryos provides the opportunity for genome-wide transcription of genes, intergenic regions, and ERV elements, such promiscuous transcription may threaten genome integrity and affect the establishment of the necessary specific gene expression pattern. Because of the inefficient splicing and 3′-processing of nascent transcripts in one-cell embryos, transcribed mRNAs, including ERV elements, are mostly nonfunctional at the one-cell stage, and this may avoid the generation of functional ERV proteins and ERV retrotransposition and facilitate the establishment of the specific gene expression pattern required for continued development [1].

tRNA-derived fragments (tRFs), originally generated in epididymis, are transferred into sperm via epididymosomes that contained tRFs and fuse with sperm during epididymal transit. Preimplantation embryos consequently acquire tRFs during natural fertilization. In two-cell embryos, tRFs are capable of repressing MERVL expression when epigenetic marks are reset to enable totipotency [74]. The tRFs that include the 3′-terminal CCA of mature tRNAs inhibit the two most active ERV families, IAP and MusD/ETn [75]. ERVs usually use the 3′ terminus of mature tRNAs as a primer that can bind the primer-binding sequence (PBS) in ERV transcripts, after which they initiate the reverse transcription process [76]. When sperm-derived 3′-terminal CCA tRFs compete with mature tRNAs for binding the PBSs in ERV transcripts, the reverse transcription process of ERV transcripts is inhibited.

The structural maintenance protein, chromosome flexible hinge domain-containing protein 1 (SMCHD1), is maternally inherited [77,78]. Previous work demonstrated that the elimination of SMCHD1 inhibited inner cell mass formation, blastocyst formation, and term development [78]. Recently, it was shown that SMCHD1 negatively regulates the *Dux* gene family, and a knockdown of SMCHD1 in zygotes results in the overexpression of DUX and ZSCAN4, accompanied by a prolonged overexpression of DUX until the eight-cell stage [79]. SMCHD1 indirectly inhibits ERV expression by regulating the *Dux* gene, which normally activates ERV elements and other ZGA-related genes. In addition, a bioinformatics analysis revealed that 89% of the ZGA-related genes are targets of either SMCHD1 or DUX, indicating that SMCHD1 can regulate the vast majority of ZGA-related genes either indirectly via DUX or directly and that SMCHD1 may facilitate the establishment of a global transcriptionally repressive state to allow exit from the ZGA state [79].

The retrotransposon long interspersed element 1 (LINE1) undergoes substantial demethylation during preimplantation development [80]. Increasing evidence has shown that LINE1 activation and expression, which are essential for preimplantation development, occur at the two-cell stage in preimplantation mouse embryos [81,82,83]. Despite the high levels of LINE1 RNA expression, the rate of LINE1 retrotransposition is low in preimplantation embryos. Further research demonstrated that LINE 1 transcripts serve as a nuclear RNA scaffold that recruits NUCLEOLIN/KAP1 to form the LINE1 RNA–NUCLEOLIN–KAP1 complex, which then represses DUX and indirectly inhibits the expression of ERVs. Conversely, LINE 1 knockdown causes prolonged DUX expression and a failure to exit from the two-cell state, ultimately resulting in embryo development retardation [84].

KRAB-ZFPs have been implicated in silencing ERVs in a sequence-specific manner. KRAB-ZFPs bind DNA through their zinc fingers and recruit the corepressor KAP1 (TRIM28, TIF1b) via their KRAB domain. In turn, KAP1 induces silencing at the target ERV locus via the recruitment of histone deacetylases, heterochromatin protein 1 (HP1), and the histone methyltransferase SETDB1 (ESET, KMT1E). For example, ZFP809, a member of the KRAB-ZFP family, is required to establish epigenetic silencing of ERVs during embryonic development. The genomic copy number of ZFPs correlates with the number of ERV LTRs, which indicates a persistent coevolution between ERVs and ZFPs [85]. The continuous cycle of KRAB-ZFP evolution against ERVs is often referred to as an evolutionary arms race [86], which may provide a driving force for new adaptations in mammals.

## 5. ERVs: Friend or Foe for Host

The strategy of expansion within germlines may be suitable for the colonization of host genomes by ERVs. In this sense, ERV expression and copying within early cleavage-stage embryos may allow ERV proliferation and expansion within host cell lineages; thus, early cleavage-stage embryos are a hotbed for retrotransposon-mediated genome evolution. In contrast, ERV copying in differentiated cells can result only in transmission between hosts, which is a proviral dead-end strategy.

ERVs use the epigenetic reprogramming window that occurs in early embryos. It may seem that early embryos tolerate a massive onslaught of ERV expression and that hosts have endured ancient retrovirus invasions for millions of years, with ERVs representing a fossil record of past viral infections. However, the activation of ERVs is not merely a relic of selfish manipulations that facilitated ERV propagation within hosts; rather, it is a formidable evolutionary force that has reshaped the host genome architecture. Retrotransposition events, which can change the size, content, and function of mammalian genomes, increase the phenotypic variability to facilitate the adaptation of host to environment. The endogenization of retrovirus sequences disrupts or alters gene expression patterns, then provides an opportunity for these genomic elements to be exploited in the context of the embryonic genome owing to the potential regulatory information carried by these retrovirus-derived sequences [87,88,89]. For example, ERVs, located in the vicinity of endogenous genes, are capable of regulating nearby genes and embryonic development via providing alternative promoters or enhancers, or orchestrating high-order chromatin assembly [47,90]. The co-opted virus-derived sequences within genomes in turn facilitate the emergence of novel adaptive cellular functions. Therefore, from an evolutionary viewpoint, the transitions from viral functions to cellular functions that have occurred repeatedly and the seemingly redundant or convergent organismal function have been established progressively [42,49]. Any host–virus interaction that can alleviate the conflict between host and virus likely promotes the fixation, retention, and diversification of virus-derived elements [91]. Therefore, when they benefit host fitness, these retrovirus-derived sequences will become fixed in and co-opted by the host, which indicates a crucial step toward normal embryonic development. Britten and Davidson have argued that the high level of ERV expression in early embryos suggests that ERV regulatory sequence exaptation occurs within species to suit their own needs [92,93]. Together, the success of ERVs lies in the commensal and even mutualistic strategy by which ERVs have evolved. This strategy produced various novel cellular functions, which in turn have promoted the cooption of ERV elements by hosts. ERV-mediated regulation of gene expression may make great contributions to the evolution and diversification of mammals. ERV–germline interactions must be mutually beneficial, such that the repression of ERVs could lower germline fitness, ensuring an ongoing motivation for ERV expression and successful host reproduction. This kind of mutually beneficial cooperativity is based on an increased genetic variability that consequently increases phenotypic plasticity and adaptability and facilitates fitness and survival within the host species [94].

We propose that ERV–germline beneficial cooperation is also a delicate compromise that is negotiated within early embryos, as evidenced by the massive upregulation of ERV expression during ZGA. Simply stated, ERVs make a compromise that their temporal and spatial expressions are tightly regulated with epigenetic modification, leaving the ZGA process as the time window for ERV activation, while the host also compromises by having early embryos allow transient ERV expression, which provides ERVs with an opportunity to spread within the host genome. There must be a delicate balance between “host defense” and “ERV escape”. Despite host silencing mechanisms, newly evolved ERVs can sometimes still escape surveillance and silence, and this applies pressure on hosts to evolve novel strategies against ERV retrotransposition. It seems that this genetic conflict promotes the rapid co-evolution of “host defense” and “ERV escape”, like an “arms race game” (e.g., the interaction between ERVs and KRAB-ZFPs) [95]. However, under selection pressure, ERV fitness ultimately depends upon the fitness of their host genomes, i.e., once ERVs increase the host fitness, they are maintained within host genomes. Specifically, because ERV expression within early embryos ensures ERV transmission and increases the number of ERV copies within host genomes, the fitness of ERVs is tightly bound to the fitness of early embryos.

## 6. The Implications of ERVs Activation for Improving the Developmental Competence of Cloned Embryos

SCNT is the process through which a donor somatic nucleus is reprogrammed by a recipient oocyte, resulting in a cloned embryo carrying the genetic information of the donor cell. Since the cloning of the first sheep, Dolly, SCNT has been successfully applied in a variety of mammalian species [30,31]. However, SCNT-mediated reprogramming is still very inefficient [31,33]. Compared with normally fertilized embryos, SCNT embryos from multiple species have a relatively high rate of arresting in the early developmental stages. For example, nearly half of mouse SCNT embryos arrest at the preimplantation stage [37,96,97,98,99]. Increasing evidence has shown that an abnormal ZGA process may be one of the main causes of developmental block in cloned embryos [33,35,36,37]. A large number of genes fail to be activated during ZGA [38]. It is now known that bursts of ERV transcription are a hallmark of ZGA [16,17,22,50,89,100]. Not surprisingly, transcriptional deregulation of ERVs during ZGA was also found in a high percentage of cloned embryos, and low expression of ERV elements in cloned embryos may correlate with ZGA failure and lower developmental competency, suggesting a tight link between the appropriate activation of ZGA-specific ERVs and the developmental potential of cloned embryos [33,34,37]. Matoba et al. also argued that the failure of *Zscan4d* activation at the two-cell stage is not the unique reason for developmental block in cloned embryos. Rather, owing to the decreased MERVL expression, gene networks regulated by ERV elements, such as MERVL, may also be defective and cause developmental block [33]. The MERVL::tdTomato reporter vector, which is under the control of the MERVL 5′-LTR, can not only reflect ERV expression patterns but also provide an indication of the expression levels of ZGA-related genes. Using this MERVL::tdTomato-based ZGA real-time monitoring system, Yang et al. found that only 12% of SCNT embryos exhibited ERV reactivation at the two-cell stage, whereas 92% of intracytoplasmic sperm injection (ICSI) embryos exhibited reactivation [37]. Together, these findings suggest that the upstream regulators of ERVs, such as DUX, may be repressed improperly. Indeed, DUX exhibited abnormal transcription in cloned mouse embryos during minor ZGA [38]. Yang et al. also found that H3K9me3, which was enriched within the *Dux* cluster of donor cells, was still not fully removed in two-cell cloned embryos and that *Dux* failed to be expressed in early two-cell cloned embryos [39]. Therefore, the aberrant DUX activity in cloned embryos during minor ZGA may explain why bursts of ERV transcription were not observed in these embryos.

The expression of *Dux* family genes is associated with the unique chromatin state of embryos during minor ZGA. It is the extremely loosened chromatin structure that permits widespread transcription from the global genome, including that of *Dux* family genes. Nevertheless, with regard to the chromatin state of one-cell embryos, differences exist between in vitro fertilization (IVF) embryos and cloned embryos. An analysis of eGFP-H2B mobility revealed that the extent of chromatin loosening is lower in cloned embryos than in IVF embryos [64], suggesting that without an open chromatin structure, somatic cell nuclei, transferred into an oocyte, may be involved in silencing *Dux*. Chromatin looseness seems to be influenced by epigenetic modifications, and heterochromatin regions where repressive epigenetic modifications, such as H3K9me3 and H3K27me3, are enriched exhibit lower histone mobility compared with euchromatic regions [64,101]. After fertilization, the male pronucleus undergoes a protamine-to-histone exchange through the incorporation of maternal histone variant H3.3. At this time, the chromatin in the male pronucleus is looser than that in the female pronucleus, which means that genome-wide transcription occurs first in the male pronucleus [64]. However, there are substantial differences in the chromatin states between SCNT-donor cells and gametes. Through a comparative transcriptome analysis, Matoba et al. identified reprogramming-resistant regions (RRRs) that are expressed normally in two-cell mouse IVF embryos but not in SCNT embryos. Although donor somatic nuclei acquire widespread chromatin remodeling during the SCNT process, RRR that are enriched for H3K9me3 may suppress the activation of genes, including *Dux* family genes [33].

**Figure 1:**
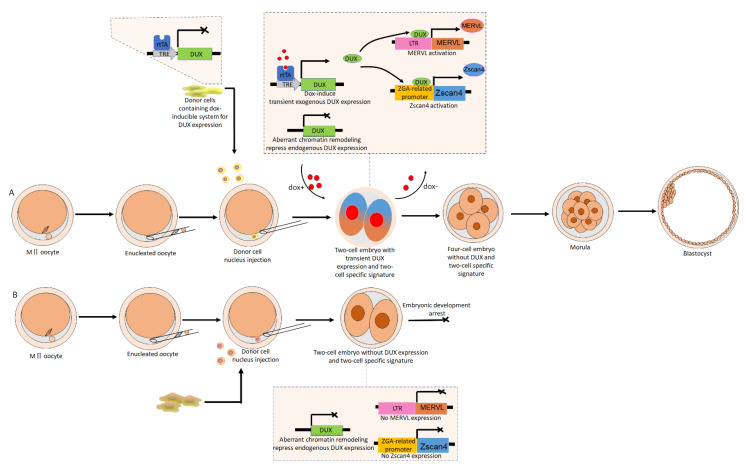
Schematic illustration showing how to induce the transient expression of exogenous double homeobox (DUX) via a dox-inducible system in cloned embryos. (**A**) Somatic cell nuclear transfer (SCNT) was performed with donor somatic cells that contained a dox-inducible system. The dox was supplied during the time window from late one-cell stage to early two-cell stage by switching between dox-containing and dox-free culture medium. With dox treatment, reverse tet-transactivator (rtTA) is activated, then DUX, controlled by tetracycline-regulated element (TRE), is expressed. Although the expression of endogenous DUX was still not initiated, owing to the aberrant chromatin remodeling in cloned embryos, the exogenous DUX was transiently expressed, and DUX activated murine endogenous retrovirus (MERVL) and Zscan4 via binding to long terminal repeat (LTR) or ZGA-related promoter, allowing the embryo to ultimately overcome its developmental arrest. Owing to the withdrawal of dox, the four-cell embryo, which lacks DUX and normal two-cell signatures, continued its own development and reached the blastocyst stage. (**B**) SCNT was performed with donor somatic cells that lacked a dox-inducible system. When the cloned embryo developed to the two-cell stage, endogenous DUX was not initiated due to the aberrant chromatin remodeling in cloned embryos, and neither MERVL nor Zscan4 were activated. The cloned embryo was arrested at the two-cell stage.

Because the timely expression of ZGA is indispensable for the development of cloned embryos, and DUX, acting as a pioneer transcription factor, can trigger the activation of ERVs and ZGA-specific genes [27,28,29], it is reasonable to suspect that exogenous DUX-treated cloned embryos will acquire higher developmental competence. Yang et al. demonstrated that a direct injection of DUX has no significant effect on SCNT blastocyst formation, whereas the transient expression of DUX indeed improves SCNT efficiency, producing a transcriptome profile similar to that of fertilized embryos [73]. The ZGA process is under the control of a “zygotic clock”, and the improvement to the efficiency of cloned embryo development induced by DUX is time-dependent. When DUX mRNA was directly injected into cloned embryos, the blastocyst formation rate did not increase, and there was an accompanying elevated fragmentation rate. Conversely, the transient expression of DUX via dox-inducible system during the 11–25 h post-activation period did increase the blastocyst formation rate. Moreover, the transient expression of DUX significantly increased the expression of ZGA-related genes, such as ZSCAN4 and MERVL [73]. The dox-inducible system is a powerful tool for initiating the transient expression of exogenous genes. This elevated SCNT efficiency benefits from the advantages of the dox-inducible system, which allows the regulation of DUX expression in a spatiotemporal-specific manner via switching between dox-containing and dox-free culture medium, therefore ensuring a timely entry into the ZGA state along with a timely exit from it (Figure 1).

In general, endogenous DUX expression disappears at the late two-cell stage in mouse embryos. When in vitro-transcribed mRNA of DUX-EGFP was injected into two blastomeres at this stage, both injected embryos were arrested at the four-cell stage with a two-cell signature. In parallel, when performing DUX mRNA injection in only one blastomere of late two-cell embryos, the injected blastomere, which was still expressing two-cell-specific markers such as ZSCAN4 and MERVL, was arrested, and the other blastomere developed normally [72], which indicates that, along with entering into the ZGA state, exiting from the ZGA state is also essential for preimplantation development and that prolonging the expression of DUX might hinder exit from the ZGA state [79]. Regarding the cytotoxicity caused by DUX [102,103], the application of a transient expression of DUX may represent a useful compromise between DUX-induced cytotoxicity and DUX-mediated totipotency. All these observations further emphasize that a timely clearing of DUX is indispensable for embryo development and support the idea that a transient expression of DUX may be an effective strategy for improving the efficiency of SCNT. Furthermore, through determining the difference in H3K9ac occupancy between IVF/ICSI and cloned embryos, aberrantly acetylated regions (AARs) have been identified, along with a global hypo-H3K9ac signal in cloned embryos [39,104]. Although Trichostatin A (TSA) treatment in cloned embryos significantly increased genome-wide H3K9ac, there were still certain regions that exhibited an aberrant H3K9ac signal; additionally, certain two-cell-specific genes, such as *Zscan4*, were not rescued in these cells. Intriguingly, because DUX motifs are enriched in AARs, the injection of full-length DUX mRNA into cloned embryos greatly restored H3K9ac occupancy in AARs, which drove two-cell-specific gene activation; thus, so the vast majority of the treated cloned embryos passed the two-cell stage. More importantly, the improvements to SCNT efficiency induced by treatment with TSA or Kdm4b were achieved via DUX activation [39]. In addition to DUX, DPPA2 and DPPA4 are both sufficient for activating MERVL by directly activating DUX; however, directly injecting DPPA2 or DPPA4 mRNA caused cloned embryos to arrest at the one-cell stage, which means that neither DPPA2 nor DPPA4 is likely suitable for use in rescuing cloned embryos from two-cell arrest [73]. Yan et al. showed that PIAS4 can catalyze the sumoylation of DPPA2, after which sumoylated DPPA2 inhibits the expression of two-cell-specific genes; therefore, the interaction between Dppa2 and small ubiquitin-like modifier-related proteins may result in sumoylated DPPA2, and injecting Dppa2 mRNA may inhibit the zygotic transcriptional program, ultimately impairing early embryo development [105].

In the case of interspecies SCNT (iSCNT), where the recipient ooplasm and donor nucleus are derived from different species, ZGA defects are considerably more pronounced. Incompatibility between the mtDNA genes and the nuclear genes encoding mitochondrial proteins disrupt the energy-making process in embryos, which is viewed as a major cause of developmental arrest [106,107]. Additionally, the very early genes that trigger autonomous transcription of the zygotic genome, including the pioneer transcription factor, should not be ignored. Given the varied histories of host exposure to exogenous retroviruses, ERV elements are diverse among different species. ERVs may be involved in the processes of molecular diversification that potentially leads to reproductive isolation, ultimately participating in the processes of speciation. We hypothesize that without species-specific DUX, the species-specific ERV elements located in donor nuclei will not be activated, in which case the ZGA process is destined to fail, and that introducing exogenous donor-specific DUX into reconstructed iSCNT embryos may alleviate ZGA defects.

## 7. Conclusions

For hosts, ERVs act as a combination of friend and foe; ERVs are genetic elements that hosts must exploit, despite needing to inhibit ERV activation during the vast majority of their life cycle. Now, in light of the indispensable roles played by ERVs during ZGA, we prefer to describe ERVs as being a “frenemy”. Apart from DUX4 and Dux, DUXC, the DUX homologs, were also observed in other Laurasiatheria, such as cow, dog, and pig. *DUX4* and *Dux* arose by retrotransposition from ancestral *DUXC* gene, and share similar homeodomain sequences and the C-terminal transcriptional activation domain with DUXC. Due to their close relatedness, DUX4 and DUXC could be functional homologs, which indicates that DUX homologs in other Laurasiatheria may also regulate ERV expression and participate in ZGA [108]. Meanwhile, we shall also bear in mind that ERVs located in genomes are species-specific, and DUX homologs have diverged in their ability to activate subsets of ERVs [28].

Unlike other existing methods of improving reprogramming efficiency by regulating epigenetic modification-related enzymes, the novel method of improving SCNT efficiency by introducing an exogenous pioneer transcription factor, such as DUX, into cloned embryos bypasses a portion of epigenetic reprogramming barriers that pre-exist in donor nuclei and dominates the activation of ERVs and two-cell-specific genes at the onset of ZGA. In the future, further dissection of the gene regulatory network, which has been rewired by ERVs, may provide insights into the mechanisms underlying ZGA, thus offering potentially novel approaches for improving SCNT efficiency.
